# Novel mechanisms for the synthesis of important secondary metabolites in *Ginkgo biloba* seed revealed by multi-omics data

**DOI:** 10.3389/fpls.2023.1196609

**Published:** 2023-06-07

**Authors:** Bing He, Kun Qian, Xin Han, Jianyang Li, Qi Zhou, Li-an Xu, Hailin Liu, Peng Cui

**Affiliations:** ^1^ Agricultural Genomics Institute at Shenzhen, Chinese Academy of Agricultural Sciences, Shenzhen, China; ^2^ Co-Innovation Center for Sustainable Forestry in Southern China, Nanjing Forestry University, Nanjing, China; ^3^ Institute of Forestry Breeding, Zhejiang Academy of Forestry, Hangzhou, China

**Keywords:** ginkgolic acids, secondary metabolites, large intron, gene expression, Ginkgo biloba

## Abstract

Although the detailed biosynthetic mechanism is still unclear, the unique secondary metabolites of *Ginkgo biloba*, including ginkgolic acids (GAs) and terpene trilactones, have attracted increasing attention for their potent medicinal, physiological and biochemical properties. In particular, GAs have shown great potential in the fields of antibacterial and insecticidal activities, making it urgent to elucidate their biosynthetic mechanism. In this study, we systematically revealed the landscape of metabolic-transcriptional regulation across continuous growth stages of *G. biloba* seeds (GBS) based on multi-omics mining and experimental verification, and successfully identified all major types of GAs and terpene trilactones along with more than a thousand kinds of other metabolites. The phenological changes and the essential gene families associated with these unique metabolites were analyzed in detail, and several potential regulatory factors were successfully identified based on co-expression association analysis. In addition, we unexpectedly found the close relationship between large introns and the biosynthesis of these secondary metabolites. These genes with large introns related to the synthesis of secondary metabolites showed higher gene expression and expression stability in different tissues or growth stages. Our results may provide a new perspective for the study of the regulatory mechanism of these unique secondary metabolites in GBS.

## 1 Introduction

As the only surviving member of the Ginkgo family, *Ginkgo biloba* is a relict plant from the Quaternary Ice Age and is widely known as the “living fossil” (Zhou and Zheng, 2003). In addition to its special evolutionary status, as a typical economic gymnosperm, *G. biloba* also possesses a variety of values, including edible, medicinal and ornamental uses. For example, a standardized preparation of *G. biloba* extract (EGB761) is among the best-selling herbal remedies in America and many European countries (Gulec et al., 2006; Nabavi and Silva, 2019). The dietary supplements sales of *G. biloba* exceed US$ 1.26 billion annually in the global market (Mei et al., 2017), and are utilized to treat thrombosis, inflammation, cardiovascular condition and Alzheimer’s disease (Sasaki et al., 2002; Rodriguez et al., 2007). In addition, *G. biloba* seed (GBS) has a very long history of consumption around the world for its important secondary metabolites, which mainly contains the medicinal substances flavonoids and terpenoids, together with the unique ginkgo phenolic acids (van Beek, 2005; Mahadevan and Park, 2008). Unlike flavonoids, which are ubiquitous in plants and have a relatively clear synthetic pathway, the research into the unique metabolites of Ginkgo has lagged behind (Ye et al., 2020; He et al., 2021).

The terpenoids in *G. biloba* are a unique class of terpene trilactones, which are composed of sesquiterpene (bilobalide) and diterpenes (ginkgolide). Ginkgolide is a potent platelet activating factor (PAF) antagonist, which can selectively inhibit platelet aggregation and prevent thrombosis, and is widely used in the treatment of cardiovascular and cerebrovascular diseases (Yang et al., 2017). Bilobalide, on the other hand, does not appear to have PAF antagonistic activity and has been shown to exert central neuroprotective effects (Atzori et al., 1993). In terms of industrial applications, currently these terpene trilactones could only be extracted directly from *G. biloba* tissues due to the unclear biosynthesis mechanism and cost limitations. Meanwhile, it should be mentioned that the terpenoid content of Ginkgo tissues is not satisfactory and could easily be affected by seasonal changes or other environmental factors, causing problems for downstream industrial preparation (Cartayrade et al., 1997).

Ginkgo phenolic acids are another group of important secondary metabolites in GBS, which are mainly concentrated in mature sarcotesta. Ginkgo phenolic acids belong to the urushiolic acids, which include three major groups of ginkgolic acids (GAs), ginkgols, and bilobols (van Beek, 2002). The GAs are referred to various n-alkyl phenolic acids with side chain length of 13-17 and side chain double bond number of 0-2, which are the main substance of ginkgo phenolic acids, accounting for 90% (Yang et al., 2014). Ginkgo phenolic acids have long been considered a typical hazardous substance due to their strong sensitization and cytotoxicity, and their proportion has been strictly limited in the preparation of ginkgo medicinal remedies. On the other hand, due to their unique hydrophobicity and hydrophilicity, ginkgo phenolic acids have been gradually applied in many fields, including antimicrobial (Lee et al., 1998), chemical (Jiang et al., 2017) and pest control (Pan et al., 2006b). Among them, GA (15:1) has been shown to prevent the acylation of small ubiquitin-related modifier proteins (SUMO) to regulate cell functions, which has been utilized as a potential treatment for cancer and neurological diseases (Fukuda et al., 2009; Baek et al., 2017).

Although some progress has been made in recent years on the anabolic regulation of important secondary metabolites in *G. biloba* (He et al., 2015; Li et al., 2018; Ye et al., 2020), most of the current research is still focused on leaves, and there is still a lack of research on the regulation of secondary metabolite synthesis in GBS considering its rich editable values. In addition, the results of systematic analysis of GAs are obviously insufficient compared to those of flavonoids and other substances. With the flourishing development of multi-omics analysis technology, new insights could be provided for the study of the *in vivo* synthesis mechanism. In this study, we aim to construct a metabolite map of GBS during the whole development period supported by these multi-omics data, and mainly focus on these unique secondary metabolites including GAs and terpene trilactones based on their detailed systematic characterization, in an attempt to better explain the specific mechanisms of their anabolism.

## 2 Materials and methods

### 2.1 Sample collection and preparation

Due to the relatively long developmental process of GBS, which takes about half a year from pollination to seed maturity, including the physiological post-ripening phenomenon. The plump seeds of a healthy female *G. biloba* tree aged about 20 years were selected and sampled every month from June to October on the campus of Nanjing Forestry University (32°4’N, 118°48’E). Samples from three representative periods (June 1, August 1, and October 1, respectively) with three replicates were selected for subsequent analysis based on the morphological observation. After sampling, these tissues were immediately frozen in liquid nitrogen and stored at -80°C until further treatment.

### 2.2 Metabolomic analysis

Biological samples were first lyophilized in a lyophilizer (SCIENTZ-100F/A) and subsequently ground (30 Hz, 1.5 min) to powder using a grinding machine. The 100 mg of powder was then weighed and dissolved in 1.0 mL of extract. The dissolved samples were refrigerated overnight at 4 °C overnight and swirled three times during this time to improve the extraction rate. After centrifugation (rotation speed: 12000 rpm, 10 min), the supernatant was extracted with the samples filtered through a microporous membrane (0.22 μm pore size) and stored in injection bottles for UPLC-MS/MS analysis.

The data acquisition system consisted mainly of an UPLC-ESI-MS/MS system (SHIMADZU Nexera X2 Series, www.shimadzu.com) and a tandem mass spectrometer (Applied Biosystems 6500 Q TRAP, www.appliedbiosystems.com). Both positive and negative ion modes were controlled by Analyst 1.6.3 software (AB Sciex). Instrument tuning and quality calibration were performed with 10 and 100 μmol/L polypropylene glycol solution in QQQ and LIT modes, respectively. QQQ scanning adopts MRM mode and sets collision gas (nitrogen) as the medium. DP and CE of each MRM ion pair were completed by further optimization, and a specific set of MRM ion pairs would be monitored at each period based on the eluted metabolites.

### 2.3 Identification and quantification of metabolites

The metabolites were characterized according to the secondary spectral information. The isotopic signals and the repetitive signals containing K^+^ ions, Na^+^ ions or ${\text{NH}}_{4}^{\;+}$ ions, and the repetitive signal of other substances of high molecular weight were removed. Metabolites were quantified by multiple reaction monitoring (MRM) using triple quadrupole mass spectrometry. In the MRM mode, the precursor ions (parent ions) of the target substance were first screened by the quadrupole, and the corresponding ions of other molecular weight substances were excluded to first eliminate the interference. After ionization induced by the collision chamber, the precursor ions would break and form many fragments. Then, a characteristic fragment ion was selected by triple quadrupole filtration to eliminate the interference of non-target ions in order to make the quantification results more accurate. After obtaining the metabolite spectrum of different samples, the mass spectrum peaks of all substances were integrated with the peak area, and these peaks identified as the same metabolite in different samples were integrated with correction.

Due to the typically high-dimensional nature of metabolomic data, the traditional principal component analysis (PCA) may not be able to cluster these data well, resulting in the poor interpretation of the analysis results. Therefore, the identification of differential metabolites was conducted with orthogonal partial least squares discriminant analysis (OPLS-DA), which combines both orthogonal signal correction (OSC) and the partial least squares discriminant analysis (PLS-DA) (Thevenot et al., 2015). In contrast to PCA, which is an unsupervised classification method, PLS-DA is a multivariate statistical analysis method with supervised pattern recognition. It could extract the components of the independent variable X and the dependent variable Y, and then calculate the correlation between each component. Based on the analysis results of the variable importance in projection (VIP) values in the OPLS-DA model, differential metabolites were screened by combining fold changes, p-values of t-test and VIP values, and the filtering standard was set as fold change > 1 and VIP > 1 with p-value should be less than 0.05.

### 2.4 Transcriptome sequencing and analysis

RNA concentration and purity were measured using NanoDrop 2000 (Thermo Fisher Scientific, Wilmington, USA, www.thermofisher.com). RNA integrity was assessed using the RNA Nano 6000 Assay Kit of the Agilent Bioanalyzer 2100 system (Agilent Technologies, CA, USA, www.agilent.com). A total amount of 1 μg RNA per sample was used as input for the RNA sample preparation. Clustering of the indexed samples was performed on a cBot Cluster Generation System using the TruSeq PE Cluster Kit v4-cBot-HS according to the manufacturer’s instructions. After clustering, library preparations were sequenced on an Illumina Hiseq X Ten platform to generate paired-end reads. Raw reads were first trimmed with fastp to remove reads containing adapter, poly-N, or low quality (Chen et al., 2018). Only reads with a unique match were further analyzed and annotated based on the reference genome (Liu et al., 2021). HISAT2 (Kim et al., 2019) was utilized to align the reads to the reference genome, and quantification of gene expression levels was estimated by fragments per kilobase of transcript per million fragments mapped (FPKM) using StringTie (Pertea et al., 2015). Differential expression analysis was performed with DESeq2 based on the negative binomial distribution (Love et al., 2014). The resulting p-values were adjusted using the Benjamini and Hochberg’s approach to control for false discovery rate (FDR) (Thissen et al., 2002).

### 2.5 Gene family analysis and functional validation

The identification of gene families related to secondary metabolite biosynthesis was performed using BLAST with e-values of 1e^-10^, and the potential members were further filtered based on the evidence of full-length transcript according to our previous data (Han et al., 2021). The Ka/Ks rate of these gene families was calculated using KaKs_calculator 2.0 (Wang et al., 2010). Weighted Gene Correlation Network Analysis (WGCNA) was utilized to infer the gene co-expression pattern (Langfelder and Horvath, 2008), and the transposable elements were identified with EDTA (Ou et al., 2019).

The same batch of collected seeds was used for the qRT-PCR experiment with three biological replicates. An RNAprep Pure Plant Kit (Polysaccharides&Polyphenolics-rich) (Tiangen Biotech, Beijing, China, www.tiangen.com) was applied to extract total RNA from each sample. PrimeScript™ RT Master Mix (Takara, Tokyo, Japan, www.takarabiomed.com) was used for reverse transcription, and the reverse transcribed product was diluted 3-fold for real-time quantitative experiments. The according primers were designed (**Supplementary File 1**) and the qRT-PCR analysis was performed using SYBR Premix Ex Taq (Takara, Tokyo, Japan, www.takarabiomed.com) and ViiA 7 Real-Time PCR System (Thermo Fisher, USA, www.thermofisher.com), and each reaction was subjected to 3 technical replicates. PCR amplification was performed under the following conditions: 95 °C for 10 min; 95 °C for 15 s, and 60 °C for 60 s with 45 cycles; 95 °C for 15 s, 60 °C for 60 s, and 95 °C for 15 s. EIF3D was set as the endogenous control, and the relative expression level was calculated using the 2^-ΔΔCt^ method.

## 3 Results

### 3.1 The landscape of GBS metabolites across continuous developmental stages

**Figure 1:**
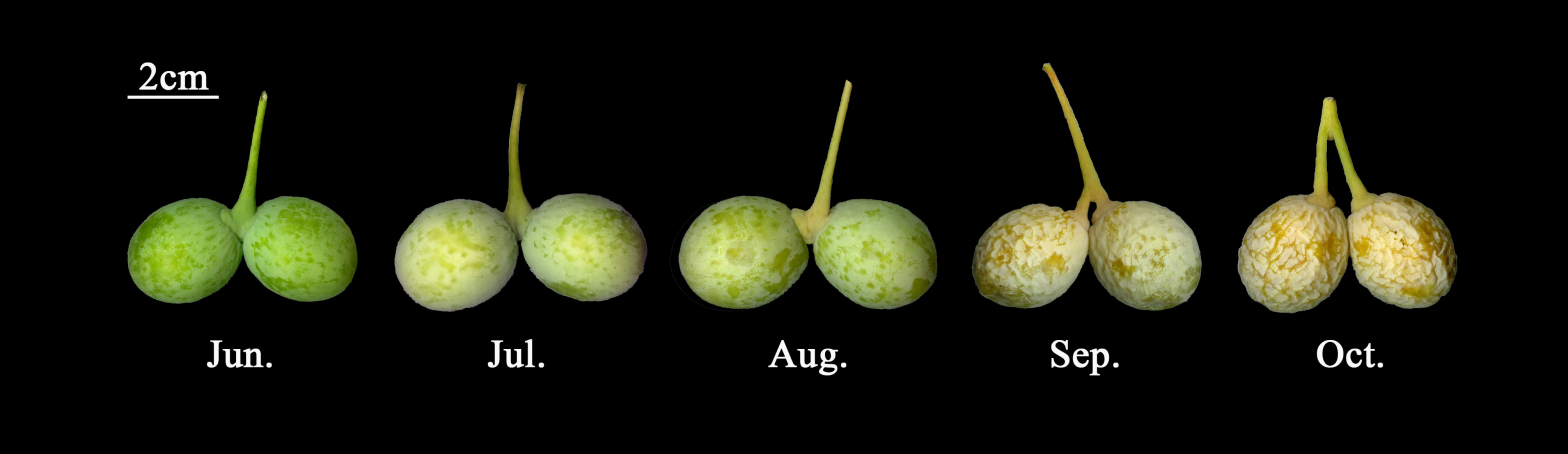
Morphological observations of the complete developmental stages of GBS.

Ginkgo seeds were sampled at three developmental stages: June, August, and October, along with the quality assessment of these samples (**Figure 1**). The correlation results of the metabolome showed that the sample results within the same batch were satisfactory, and the results of the principal component analysis (PCA) were able to fully reveal the significant differences between samples from different batches. The transcriptome results were also in line with our expectations. These results demonstrated the robustness of the data quality, which could strongly support our subsequent analysis.

**Figure 2:**
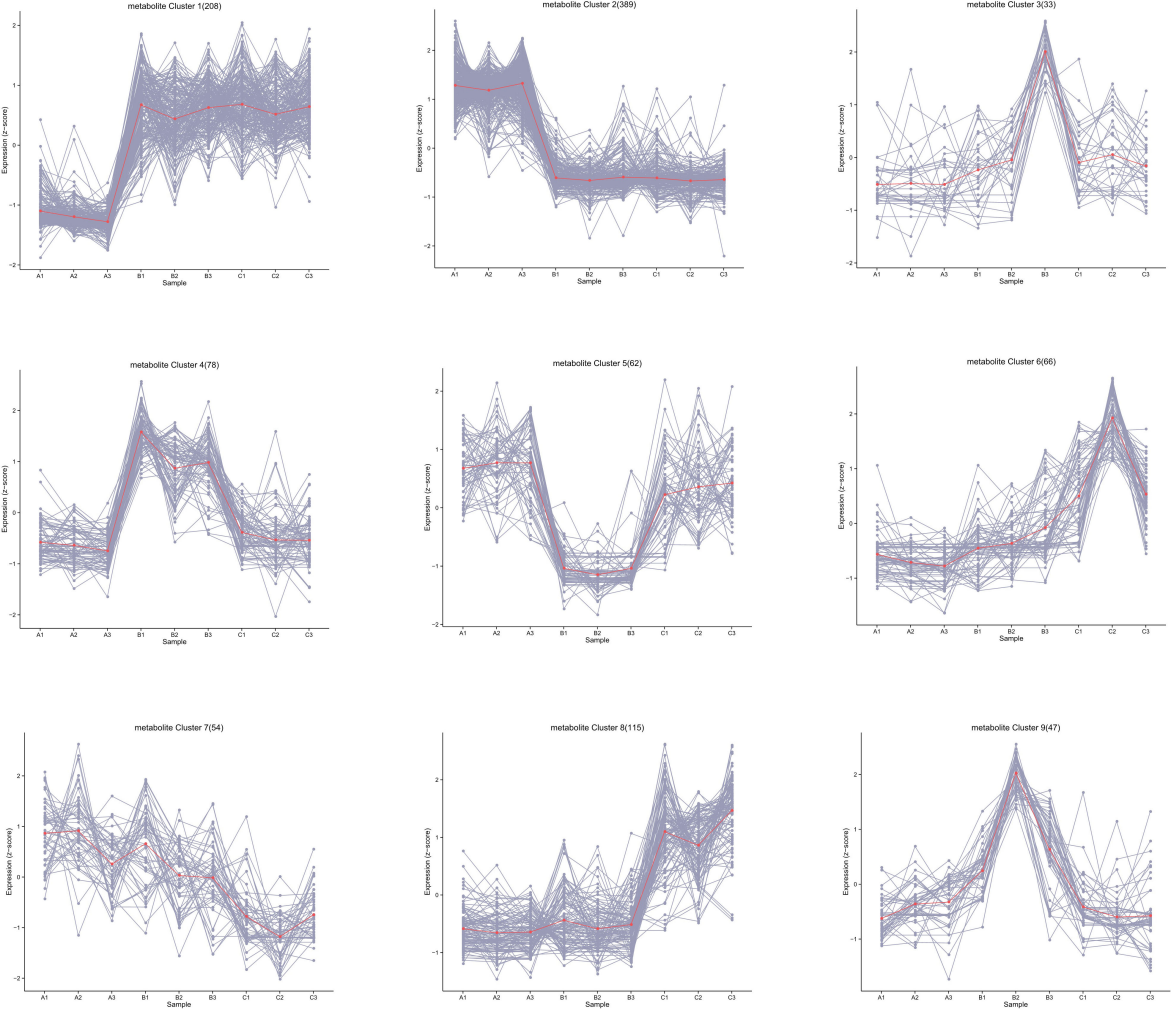
Classification of the trend changes in the content of all metabolites over different time periods.

**Figure 3:**
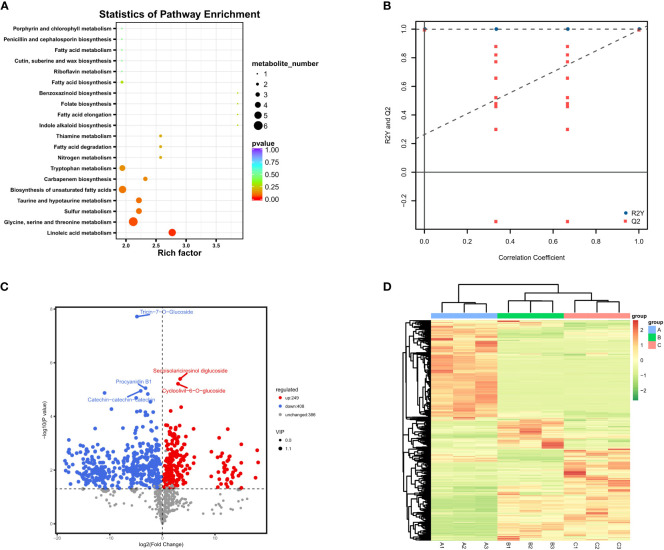
Differential metabolite results; **(A)** Annotated bubble diagram of metabolites for Group 1; **(B)** Permutation plot of the OPLS-DA model. The horizontal axis indicates the similarity to the original model, and the vertical axis indicates the value of R2Y or Q2. The blue and red dots represent R2Y and Q2 of the model after Y replacement, respectively, and the dashed line is the fitted regression line. If R2Y and Q2 are smaller than R2Y and Q2 of the original model, then the model could be screened for differential metabolites according to VIP values; **(C)** Volcanic map of differential metabolites between June and August. The horizontal coordinate represents the fold change of each substance compared in this group, and the size of the scatter point represents the VIP value generated from the OPLS-DA model; **(D)** Heatmap of all metabolites based on VIP values. **(A–C)** represent June, August and October, respectively.

A total of 1,043 kinds of metabolites were detected, and the number was far more than 780 kinds obtained from *G. biloba* leaves during the same sampling periods, showing vigorous metabolic activity (He et al., 2021). The substances detected were mainly primary metabolites, including carboxylic acids and derivatives, fatty acids, and related oxidation compounds. All metabolites were then clustered into nine groups according to their expression (**Figure 2**). The results showed that most of the metabolites in ginkgo seeds were more active in June and August, and 208 of them (Group 1) significantly increased from June to August and maintained until October, while the expression levels of other 389 metabolites (Group 2), on the contrary, decreased from June to October. The number of metabolites between these two groups with opposite expression trends accounted for more than half of all metabolites, and their functional enrichment results also showed differences. Metabolites of Group 1 with obvious up-regulated expression trend were mainly enriched in amino acid and lipid metabolism, including biosynthesis of unsaturated fatty acid biosynthesis and linoleic acid metabolism together with glycine, serine and threonine metabolism, showing an active tendency in energy metabolism and storage (**Figure 3A**). On the other hand, metabolites of Group 2 with a downward tendency were mainly enriched with flavonoid biosynthesis, which was another class of important secondary metabolites in *G. biloba*.

Subsequently, the differential metabolites were screened by combining the fold changes and VIP values generated by the OPLS-DA model to further observe the content change of metabolites in different developmental stages (**Figure 3B**). From June to August, the number of down-regulated metabolites was much higher than that of up-regulated metabolites, and the levels of other 386 metabolites were relatively stable. One of the most significantly up-regulated substances during this period was turanose (log2 (fold change) > 18), which is an isomer of sucrose, and the most representative down-regulated metabolites were myricetin-3-O-glucoside and tricin-7-O-glucoside (**Figure 3C**). Tricin is biosynthesized as a component of plant secondary metabolites through a combination of phenylpropanoid and polyketide pathways, and it has been extensively studied due to its biological significance in plant growth as well as its potential for pharmaceutical importance (Zhou and Ibrahim, 2010). Both this metabolite and myricetin-3-O-glucoside belong to the flavonoid group, and their content changes were consistent with other typical flavonoids within these two periods, reaching the peak in June and dramatically decreasing in August.

According to our results, there seemed to be a significant decrease in metabolite activity and a steady state between August and October compared to the previous two months. Among them, most metabolites showed no significant change, and other 167 and 126 metabolites were up- and down-regulated, respectively (**Figure 3D**). This statistic was slightly different from the previous clustering results based on simple trend analysis, which mainly involved distinguishing the significance of the difference. The most representative metabolite, methyl cinnamate, which is an ester within the cinnamate family (Bhatia et al., 2007), was up-regulated throughout August and October with the highest fold change value. It is worth mentioning that the most significantly up-regulated metabolites during this period were also highly enriched in the phenylpropanoid biosynthetic pathway, which is involved in the synthesis of various terpenoids, including trans-cinnamaldehyde and p-coumaric acid. In particular, there seemed to be significant differences in metabolic activity at different developmental stages in GBS. The primary metabolic activity was more vigorous from June to August, while the metabolic activity tended to be stable after August and gradually accumulated secondary metabolites including terpene trilactones.

### 3.2 Identification of the unique secondary metabolites in *G. biloba*

**Figure 4:**
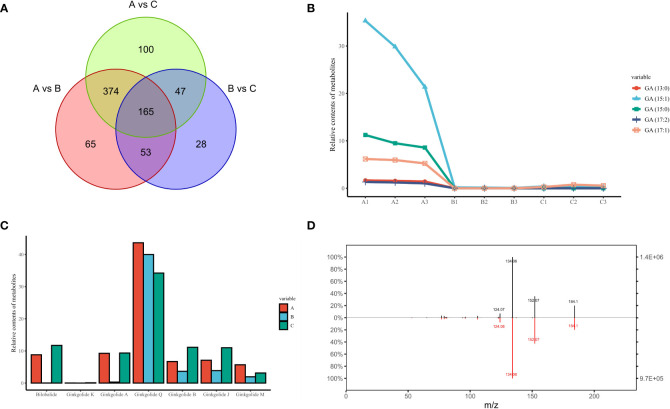
The unique secondary metabolites identified in GBS; **(A)** Venn diagram of all differential metabolites in three time periods; **(B)** Content variation of five representative GAs in three time periods; **(C)** Content variation of all types of terpenoids unique to GBS in three time periods; **(D)** Secondary spectrum of ginkgotoxin and its mirror image of the standard. The upper and lower plots represent the secondary spectrum extracted from the standard and the sample, respectively. The vertical axis represents the mass charge ratio (m/z).

From a continuous developmental perspective, June to August produced many more differential metabolites compared to August to October, and these content differences appeared to persist over a long period of time, culminating in 165 differential metabolites being present in the comparative results for any two time points (**Figure 4A**). As the unique secondary metabolites identified in *G. biloba* with potential sensitization, mutagenicity and strong cytotoxicity, all major ginkgo phenolic acids were successfully mined in our research based on their differences in side chain length and double bond number, including GA (13:0), GA (15:0), GA (15:1), GA (17:1) and GA (17:2). The chemical structures of these metabolites are similar to urushic acid in *Pelargonium hortorum*, and it is inferred that the more double chains on the hydrocarbon group, the more susceptible to sensitization (Narnoliya et al., 2017). Among them, GA (15:1) accounted for more than 60% of the total, and the content of GA (17:2) was the lowest (**Figure 4B**). According to the previous clustering results of ginkgo metabolites, the expression trends of all GAs were similar. The levels of GAs peaked in June and then decreased rapidly without any obvious fluctuation since then, which was also consistent with the results of other Group 2 metabolites.

In contrast to the hazardous properties of GAs, the unique terpene trilactones are widely used in the treatment of human cardiovascular diseases. Our results were also fruitful for the identification of terpene trilactones in GBS. A total of seven types of terpene trilactones were identified, including six typical diterpenes (Ginkgolide A/B/J/K/M/Q, respectively) and one sesquiterpene (bilobalide). Interestingly, the content variation of terpene trilactones showed a clear difference when compared to GAs (**Figure 4C**). According to the previous clustering results, all GAs shared a consistent tendency to be classified in Group 2, while different groups were confirmed among these terpene trilactones. Specifically, Ginkgolide A and bilobalide were in Group 5 with other 60 metabolites, and the typical characteristic of this group was the double peak phenomenon, which had a much higher content in June and October and decreased dramatically in August. On the other hand, Ginkgolide B、J and K were in Group 8, and the change trend of this group was completely opposite to that of Group 2. The contents of these terpenoids didn’t change much in the early months and gradually reached the peak around October. These results indicated the complexity of the terpene trilactone biosynthesis in GBS, and there seems to be a potential antagonistic relationship in their synthesis processes, although these terpenoids share common precursors.

In addition to the various GAs and terpene trilactones, the unique neurotoxin in GBS, ginkgotoxin (4′-O-methylpyridoxine, MPN), was also observed (**Figure 4D**). Ginkgotoxin is an antivitamin which is structurally related to vitamin B6 (pyridoxine), and excessive consumption of GBS could easily lead to poisoning, causing drowsiness, convulsions and seizures, loss of consciousness and even death (Leistner and Drewke, 2010). It is an inhibitor of pyridoxal kinase and glutamic acid decarboxylase in mouse brain, and its toxic effect could be alleviated by pyridoxine (Kobayashi et al., 2015). In our study, the variation trend of ginkgotoxin content was consistent with that of GAs, which reached the highest content in June and then decreased rapidly without any significant fluctuation.

### 3.3 Multi-omics analysis of GA synthesis revealed active involvement of large introns

**Figure 5:**
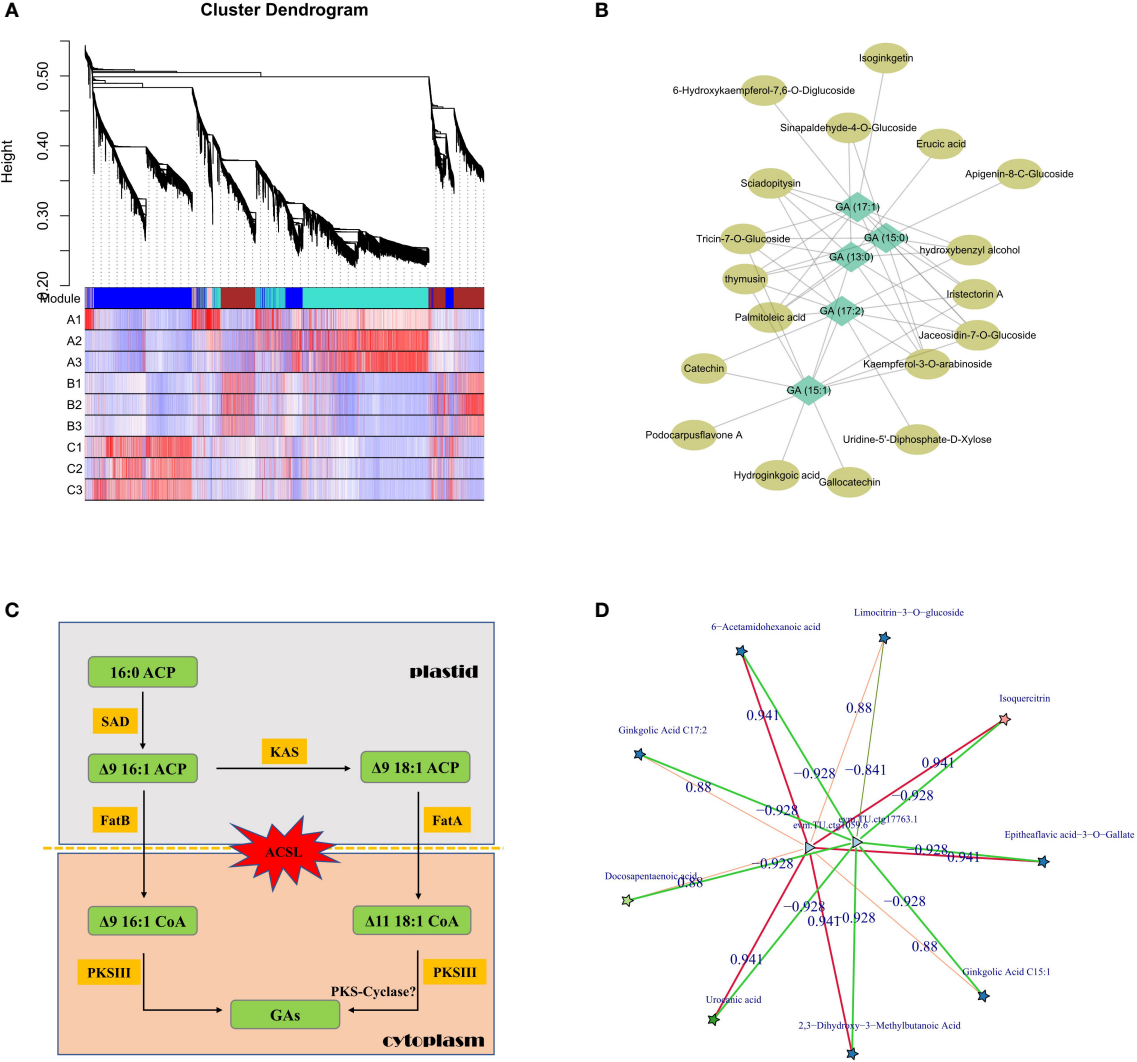
Regulation of GA synthesis in GBS; **(A)** Clustering and co-expression results of all metabolites based on WGCNA method; **(B)** Correlation network diagram of GAs and other representative metabolites; **(C)** Inferred pathway and related genes involved in GAs synthesis in plastid and cytoplasm. Genes of the ACSL family, which are responsible for transmembrane transport, underwent a significant intron length expansion; **(D)** Co-expression network of candidate essential genes involved in the regulation of GA synthesis based on transcriptome and metabolome. Stars represent metabolites and triangles represent genes. The red and green lines represent positive and negative correlations, respectively.

Although metabolome analysis could intuitively obtain the atlas and content trends of all metabolites in GBS, the combination analysis of transcriptome and other multi-omics data is necessary to reveal the detailed synthesis and regulation mechanisms. Co-expression analysis was first performed based on the relative content of values with WGCNA method, and nearly 400 metabolites were grouped together with all GAs into the same module, which was very similar to the previous clustering results in Group 2 (**Figure 5A**). Then, all metabolites in this module were further annotated and a correlation network was constructed according to their correlation weight in order to identify the essential metabolites related to GA synthesis. According to our results, palmitoleic acid and erucic acid, which are considered as the primary precursors of GAs, were found to be closely related metabolites along with several flavonoid derivatives (**Figure 5B**). Subsequently, we mainly focused on metabolic pathways for further analysis using homologous comparison and literature mining. According to previous studies, the aromatic ring and long-chain alkyl/alkenyl group of GAs are thought to be synthesized by steps that could be divided into two parts: 1. the synthesis of free palm-oleyl CoA and oleyl CoA generated from malonyl CoA/acetyl CoA based on fatty acid synthesis; 2. the synthesis of GAs from long-chain oleyl CoA using polyketides (Gellerman et al., 1976).

The synthesis of GAs is based on palmitoleic acid and oleic acid, which initially contain the necessary alkyl side chains, and the levels of both metabolites were significantly altered in our study. In this process, acyl carrier protein (ACP) is the center of fatty acid synthesis as an intermediate conserved carrier, and stearoyl-ACP desaturase (SAD) protein is a key rate-limiting enzyme in this process. It is a homodimer composed of conserved domains belonging to the ferritin family, and is the only known soluble desaturase group in plants which could regulate the ratio of saturated fatty acids (Knutzon et al., 1992). According to our results, six genes encoding SAD protein were identified in GBS, and analysis of their expression patterns showed that four of them had significantly higher expression in June than in August, while the other two genes remained relatively stable. Association analysis with the metabolome further revealed that one of them was highly positively correlated with GA (15:1) and GA (17:2) content changes. Beta-ketoacyl-ACP synthase (KAS) is another important enzyme in this process, and it is the core element of the fatty acid synthase (FAS) complex, which could catalyze the condensation of malonyl-ACP and palmitic acid to long-chain fatty acids (Mindrebo et al., 2020). A total of nine genes encoding for KAS were identified in GBS, and two of them showed a significant correlation with the variation in GA content. Chromosomal mapping of these genes revealed that four of them were distributed in obvious clusters on chromosome 6, and one of them was not only an important regulator of GA synthesis, but also highly correlated with the synthesis of other metabolites, such as isoquercitrin, which is a typical compound of flavonoids.

The next key step in the synthesis of GAs is the formation of the phenol-lipid benzene ring. After obtaining the main long-chain fatty acid precursor, several condensation reactions would occur to generate different types of GAs, and type III polyketide synthase protein (PKS III) is the key rate-limiting enzyme. PKS III is characterized by its ability to reuse homologous proteins and to react directly with acyl-CoA substrates independently of ACP activation (Yu et al., 2012). Many members have been identified in this family, of which chalcone synthase (CHS), stilbene synthase (STS), and FAE1-type keto-acyl CoA synthase (KCS) are their typical representatives. Chalcone is an important precursor of flavonoid synthesis and the CHS family is widely distributed in plants, while STS is highly homologous to CHS with amino acid sequence similarity of nearly 70% and could form resveratrol from coumaryl CoA (Schroder and Schroder, 1990). KCS is a rate-limiting enzyme that catalyzes the first step condensation reaction in the synthesis of very long chain fatty acids (VLCFAs), and the studies on the KCS family have been mainly in model plants with few studies in other species (Sagar et al., 2015). According to our results, a total of 31 genes encoding PKS III were identified in GBS, including 15 genes encoding KCS, and several of them had the consistent expression pattern with GAs. Surprisingly, although previous studies on the synthesis of phenolic acids in other species showed that PKS-Cyclase might play a key role in the formation of phenyl rings, and then catalyze the reaction between hydrogen ions and ketone groups similar to the aldol condensation in order to form double bonds (Nakano et al., 2009), not even one gene encoding PKS-Cyclase was found in GBS according to the homology comparison in this study (**Figure 5C**).

In addition to analyzing the key genes and enzymes of GA synthesis based on metabolic pathways, other associated regulatory factors were also investigated based on the co-expression network. Through rigorous screening, two differentially expressed genes (DEGs) were identified that were most highly correlated with GAs (**Figure 5D**). Functional annotation of these two genes showed one putative annotation for GDSL esterase, and the other one had an unknown annotation. The encoded protein of this unannotated gene showed very low conservation with other species, indicating that it should be a Ginkgo-specific gene involved in GA synthesis. Lipase is one of the key enzymes in seed oil degradation, which includes GDSL esterase because of its highly conserved glycine (G)-aspartic acid (D)-serine (S)-leucine (L) motif at the N-terminus of the protein. GDSL esterases are widely distributed in plants with diverse members, but only a few of them have been identified with clear molecular functions (Lai et al., 2017). A study in *Arabidopsis* has confirmed that several GDSL esterases could alter the fatty acid composition of seeds by acting downstream of the gibberellin signaling pathway, which is also consistent with our results (Chen et al., 2012).

**Figure 6:**
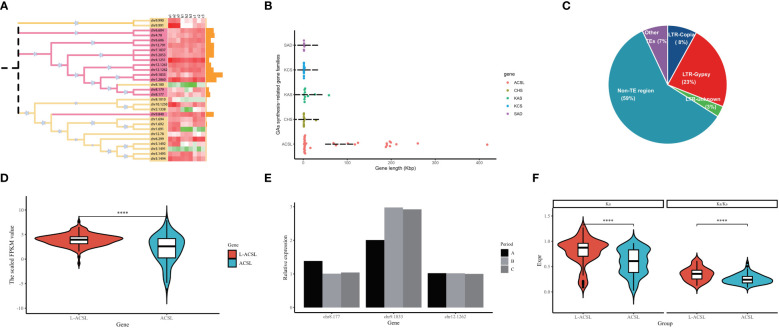
Analysis results of the ACSL gene family in GBS; **(A)** Phylogenetic tree and expression pattern of all ACSL gene family members in GBS. The heatmap represents the FPKM values of all samples in three time periods, and the bar chart represents the length of each gene; **(B)** Length distribution of *GbACSL* and other GA synthesis-related gene family members; **(C)** Detailed composition of the intron regions of *GbACSL* gene members; **(D)** Violin plot of the expression of *GbACSL* genes with and without large introns; **(E)** Expression results of *GbACSL* genes with large introns validated by RT-PCR experiments; **(F)** Ka/Ks results for *GbACSL* genes with and without large introns. **** p value < 0.0001.

Fatty acids from exogenous or endogenous sources must first be activated to form acyl-CoA before entering most metabolic pathways. This activation step is catalyzed by acyl-CoA synthase (ACS) through a two-step reaction involving the formation of the intermediate fatty acyl-AMP with the release of pyrophosphate, and the formation of fatty acyl-CoA to release AMP (Mashek et al., 2004). In *G. biloba*, nearly 30 members have been identified encoding ACSs distributed on different chromosomes, including the long-chain ACSs (ACSL) that preferentially activate the most abundant fatty acids including GAs (12-20 carbons) (**Figure 6A**). Surprisingly, more than 50% of the *GbACSL* genes in GBS were extremely long, with the average length of all ACSL genes reaching 73 Kbp and the longest even reaching 418 Kbp. The length of these genes was much longer than other genes related to GA synthesis, whose average length was less than 3 Kbp (**Figure 5B**). Further analysis showed that introns instead of exons were surprisingly elongated among these genes, and transposable elements might play an important role in their intron elongation. Meanwhile, it should be mentioned that these non-TE regions also accounted for a large proportion, which might be composed of some other decayed elements (**Figure 6C**). Interestingly, the integration analysis results showed that these large introns did not seem to impose a burden on the transcription of these *GbACSL* genes, and on the contrary, the expression of these genes was significantly increased (**Figure 6D**). In addition, the expression patterns of these large genes appeared to be more stable, maintaining a conserved expression pattern and largely unaffected by period changes, as further confirmed by subsequent qRT-PCR experimental validation of our RNA-seq results (**Figure 6E**).The selection pressure analysis results of these *GbACSL* genes were consistent with the expression patterns, and genes with large introns tended to have higher Ka/Ks values for positive selection (**Figure 6F**).

### 3.4 Co-expression analysis for precursors of terpene trilactone synthesis in GBS

**Figure 7:**
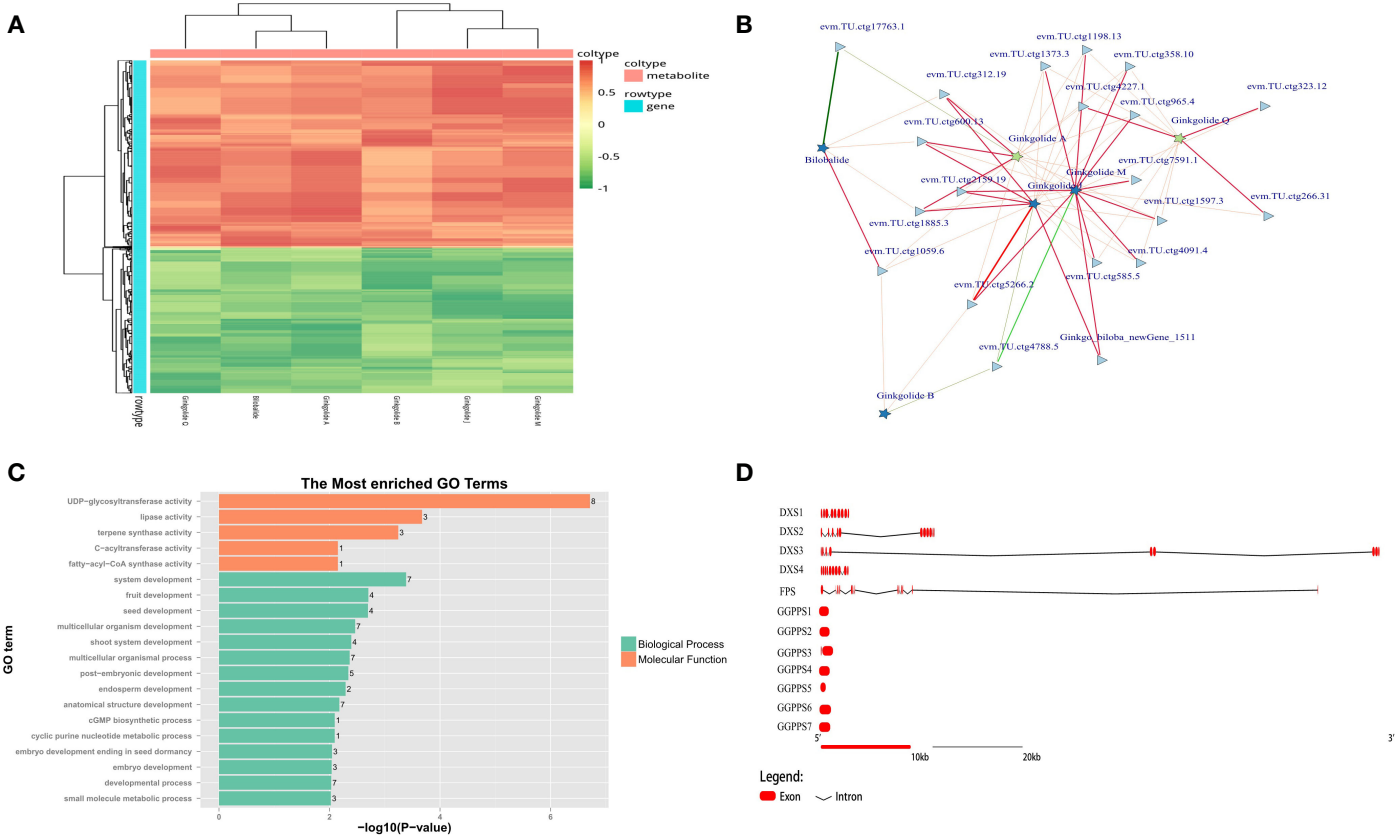
Mining results of terpene trilactone co-expression data in GBS; **(A)** Co-expression heatmap of the terpene trilactones identified in GBS and all genes; **(B)** Association network of ginkgo terpene trilactones and the essential genes based on Spearman’s correlation coefficient. The red and green lines represent positive and negative correlation, respectively, and the width of the lines indicates the strength of the correlation; **(C)** Functional annotation results of the extracted essential genes closely related to terpenoid synthesis in GBS; **(D)** Gene structure diagram of the genes involved in terpene trilactone synthesis in GBS.

According to the co-expression results based on metabolome and transcriptome, all types of unique terpene trilactones identified in GBS were successfully correlated with the gene expression profile, except for Ginkgolide K due to its extremely low content (**Figure 7A**). Two major biosynthetic pathways of terpene trilactones in *G. biloba* could be identified, including the mevalonate pathway (MVA) in the cytoplasm and the 2-C-methyl-D-erythritol-4-phosphate pathway (MEP) in the plastid. It is generally concluded that the biosynthesis of various ginkgolides is mainly through the MEP pathway, while the biosynthesis of the sesquiterpene (bilobalide) is achieved through the MVA pathway, or it may be the degradation product of ginkgolides (Penuelas and Munne-Bosch, 2005). HMGR (3-hydroxy-3-methylglutaryl coenzyme A reductase) is the first rate-limiting enzyme in the MVA pathway of terpenoid synthesis. It could catalyze 3-hydroxy-3-methylglutaryl-CoA to obtain mevalonate, which is an important precursor of terpenoid synthesis. Previous studies showed that the gene family encoding HMGR in other plants was generally small (Kim et al., 2014). Meanwhile, more than 10 members of the HMGR family were identified in GBS based on homology comparison, which was also the largest number of gene families related to terpenoid synthesis in GBS. Most of these genes were distributed in a typical cluster arrangement on chromosome 9 and chromosome 4. On the other hand, despite the large number of members, only a few HMGR genes were significantly positively correlated with terpene trilactone levels in both periods based on the results of co-expression analysis (**Figure 7B**). DXS (1-deoxy-D-xylulose-5-phosphate synthase) is the first enzyme in the MEP synthesis pathway. It catalyzes pyruvate and glyceraldehyde-3-phosphate to synthesize 1-deoxy-D-xylulose-5-phosphate, and also plays a critical role in terpenoid metabolism as a major rate-limiting enzyme (Estevez et al., 2001). In GBS, four genes encoding DXS were identified, one of which showed a high and stable expression level. Similar to the results of *GbACSL*, this highly expressed gene also had a very long length of 122 Kbp as well, including two large introns with the length of 48 Kbp and 70 Kbp respectively, which was the longest one among all rate-limiting genes involved in the synthesis of ginkgo terpenoids. Then, these highly associated genes obtained from previous co-expression predictions were further annotated, and the results showed that these genes seemed to be closely associated with seed and embryo development in addition to their active involvement in terpene biosynthesis (**Figure 7C**).

Farnesyl diphosphate synthase (FPS) is another key enzyme in this pathway with two aspartic acid-rich regions. It catalyzes the condensation reaction of two molecules of isopentenyl pyrophosphate (IPP) and one molecule of dimethylallyl pyrophosphate (DMAPP) to (2Z, 6E)-farnesyl diphosphate, which could provide the C15 scaffold for sesquiterpenoid formation (Estevez et al., 2001). Although only one gene encoding FPS was identified, this large gene length of more than 109 Kbp still maintained high expression. The corresponding enzyme of FPS is geranylgeranyl diphosphate synthase (GGPPS) in the MEP pathway, which could catalyze three molecules of IPP and one molecule of DMAPP to form geranylgeranyl diphosphate (GGPP), providing the C20 backbone for diterpenoid formation (Schmidt and Gershenzon, 2007). GGPPS is not only involved in the synthesis of ginkgolides, but also closely related to the synthesis of chlorophyll, carotenoids and gibberellins. Seven members encoding GGPPS were identified, and none of them was a large gene with an average length of less than 2 Kbp and an intronless structure (**Figure 7D**). According to our results, there seemed to be a delicate balance between the number and the length of genes in GGPPS and FPS that shared similar functions.

## 4 Discussion

As a unique secondary metabolite of *G. biloba*, the rich content of ginkgo phenolic acids in GBS is difficult to ignore for their strong sensitizing toxicity and cytotoxicity. Due to the significant antibacterial properties of GAs, including the inhibition of a variety of gram-negative and positive bacteria, rice blast fungus and other fungi, as well as the inhibition of aphids, cabbage caterpillars and other insects, GAs have the potential to be developed as biological pesticides (Muroi and Kubo, 1996; Pan et al., 2006a). Meanwhile, compared with flavonoids in *G. biloba* with clear pharmacological activities, the biosynthesis and regulatory mechanism together with the derivative efficacy of GAs have not been thoroughly studied, which severely limits their effective utilization. More than 30,000 tons of the GBS testa would be discarded each year during the production process of Ginkgo-related remedies, which not only pollutes the environment but also causes a great waste of resources (Boateng, 2022). The unique terpenoids of *G. biloba* also had the same doubts about the detailed biosynthesis and regulation mechanisms. The synthesis of terpene trilactones in GBS involves oxidation, cyclization, and rearrangement reactions, resulting in their complex structures, making their analysis lag behind that of other metabolites (Ding et al., 2008).

In this study, based on multi-omics analysis and functional validation, we systematically analyzed the phonological changes and synthetic pathways related to GAs and terpenoids in GBS, focusing on their major rate-limiting enzyme families. Analysis of the expression patterns and chromosome mapping of these genes further revealed that most of them were arranged in clusters, and the role of large introns in their expression should be paid more attention. Recent studies have shown that cytochrome P450 (CYP450) enzymes may play an important role in the downstream synthesis of terpene trilactones (Zheng et al., 2019). According to the homologous alignment results, a very large and complex group of CYP450 family and the complete loss of PKS-Cyclase in GBS suggested that there might be a unique aldol condensation reaction in the downstream synthesis process of GAs.

The heterogeneous distribution of large genes was observed during the analysis of genes related to secondary metabolite synthesis in GBS, and the enrichment was most significant in the *GbACSL* gene family. The length of these large genes exceeded the range of most plant genes, and further analysis revealed that intron elongation played an essential role. Combined with the existing features of the Ginkgo genome, a considerable number of LTR-RTs insertion events were identified in *G. biloba* and other gymnosperm genomes, which may provide some explanation for the dramatic increase in the length of Ginkgo introns (Liu et al., 2021). On the other hand, it is worth considering whether these intron-lengthening genes were simply randomly generated by violent transposable element insertions. From the point of view of energy consumption, the retention of large introns means the additional loss of energy during replication and transcription. For a large 100 Kb intron, it would be a million times harder to bring its ends together than for a 1 Kb intron in length, and a stretched 100 Kb RNA could even expand to a size larger than that of the mammalian nucleus (Shepard et al., 2009). It could be easily deduced that introns should have several advantages to compensate for these costly disadvantages, and the current results clearly showed that the significant increase in intron size is not meaningless and would lead to a stable high expression during transcription in GBS. Moreover, compared with Arabidopsis and other angiosperms, no whole genome duplication (WGD) event could be identified in Ginkgo or other gymnosperms, although this event should be a cornerstone of genome evolution in angiosperms (Clark and Donoghue, 2018), and genes with large introns in GBS showed functional enrichment along with higher positive selection pressure. These results suggest that large genes in *G. biloba* may tend to acquire their own introns independently, and the proportion of large introns in these families may be an effective indicator of their preference for environmental adaptation.

Combined with our current results, we believe it’s very possible that these large genes may further promote the growth or environmental adaptation of *G. biloba*. As for the typical large *GbACSL* gene family in this study, due to the key role of lipids in cell growth and energy metabolism, higher expression of this family, which is responsible for the acetylation of all C12-20 members of lipids, may not only be involved in the precursor synthesis of GAs, but also plays an essential role in the formation of plant signal transducers and other lipid-related molecules. Another typical example is the GGPPS and FPS gene families in terpenoid synthesis. These two families have similar functions and both use IPP and DMAPP as substrates, and the emergence of large genes seems to lead these two families to different paths in the selection of gene length and gene number. On the other hand, how to explain the significant positive correlation between these large introns and gene expression remains an unresolved doubt. We speculated that one possibility is the high-frequency occurrence of intron-mediated enhancement (IME), which has also been observed in Arabidopsis and several other plants (Gallegos and Rose, 2015). Meanwhile, it should be mentioned that these enlarged introns had no particular positional preference in the ginkgo genes, whereas IME usually occurs at the first intron, suggesting that a novel mechanism may regulate gene expression in *G. biloba*, and more multi-omics data would be obtained in our subsequent work to unravel this mystery.

## Conclusion

In this study, the global metabolite landscape of GBS throughout the almost complete growth cycle was revealed by UPLC-MS/MS platform, and the anabolic regulation mechanism of the unique GAs and terpene trilactones in GBS was systematically analyzed by integrating metabolomics, transcriptomics and other multi-omics data. Most members of the key rate-limiting enzymes within the synthetic pathways of these secondary metabolites were successfully identified along with several important regulatory factors. In addition, large introns seemed to play a very important role in the expression of genes related to secondary synthesis and metabolism in GBS.

## Data availability statement

The original contributions presented in the study are publicly available. This data can be found here: NCBI Bioproject, accession PRJNA973135.

## Author contributions

PC and HL conceived and designed the experiments. BH and XH analyzed the data and wrote the paper. KQ and JL performed the experimental validation and data calibration. L-AX modified the manuscript and QZ curated the experimental validation data. All authors contributed to the article and approved the submitted version.

## Supplementary material

Supplementary File 1 The primer information of related genes for qRT-PCR.

## References

[B1] Atzori C. Bruno A. Chichino G. Bombardelli E. Scaglia M. Ghione M. (1993). Activity of bilobalide, a sesquiterpene from ginkgo biloba, on pneumocystis carinii. Antimicrob. Agents Chemother. 37, 1492–1496. doi: 10.1128/AAC.37.7.1492 8363381 PMC188000

[B2] Baek S. H. Lee J. H. Kim C. Ko J. H. Ryu S. H. Lee S. G.. (2017). Ginkgolic acid c 17:1, derived from ginkgo biloba leaves, suppresses constitutive and inducible STAT3 activation through induction of PTEN and SHP-1 tyrosine phosphatase. Molecules 22 (2), 276. doi: 10.3390/molecules22020276 28208828 PMC6155672

[B3] Bhatia S. P. Wellington G. A. Cocchiara J. Lalko J. Letizia C. S. Api A. M. (2007). Fragrance material review on methyl cinnamate. Food Chem. Toxicol. 45, S113–S119. doi: 10.1016/j.fct.2007.09.077 18037211

[B4] Boateng I. D. (2022). A critical review of current technologies used to reduce ginkgotoxin, ginkgotoxin-5'-glucoside, ginkgolic acid, allergic glycoprotein, and cyanide in ginkgo biloba l. seed. Food Chem. 382, 132408. doi: 10.1016/j.foodchem.2022.132408 35176549

[B5] Cartayrade A. Neau E. Sohier C. Balz J. P. Carde J. P. Walter J. (1997). Ginkgolide and bilobalide biosynthesis in ginkgo biloba .1. sites of synthesis, translocation and accumulation of ginkgolides and bilobalide. Plant Physiol. Bioch 35, 859–868.

[B6] Chen M. Du X. Zhu Y. Wang Z. Hua S. Li Z.. (2012). Seed fatty acid reducer acts downstream of gibberellin signalling pathway to lower seed fatty acid storage in arabidopsis. Plant Cell Environ. 35, 2155–2169. doi: 10.1111/j.1365-3040.2012.02546.x 22632271

[B7] Chen S. F. Zhou Y. Q. Chen Y. R. Gu J. (2018). Fastp: an ultra-fast all-in-one FASTQ preprocessor. Bioinformatics 34, 884–890. doi: 10.1093/bioinformatics/bty560 30423086 PMC6129281

[B8] Clark J. W. Donoghue P. C. J. (2018). Whole-genome duplication and plant macroevolution. Trends Plant Sci. 23, 933–945. doi: 10.1016/j.tplants.2018.07.006 30122372

[B9] Ding S. Dudley E. Song Q. Plummer S. Tang J. Newton R. P.. (2008). Mass spectrometry analysis of terpene lactones in ginkgo biloba. Rapid Commun. Mass Spectrom 22, 766–772. doi: 10.1002/rcm.3424 18275095

[B10] Estevez J. M. Cantero A. Reindl A. Reichler S. Leon P. (2001). 1-Deoxy-D-xylulose-5-phosphate synthase, a limiting enzyme for plastidic isoprenoid biosynthesis in plants. J. Biol. Chem. 276, 22901–22909. doi: 10.1074/jbc.M100854200 11264287

[B11] Fukuda I. Ito A. Hirai G. Nishimura S. Kawasaki H. Saitoh H.. (2009). Ginkgolic acid inhibits protein SUMOylation by blocking formation of the E1-SUMO intermediate. Chem. Biol. 16, 133–140. doi: 10.1016/j.chembiol.2009.01.009 19246003

[B12] Gallegos J. E. Rose A. B. (2015). The enduring mystery of intron-mediated enhancement. Plant Sci. 237, 8–15. doi: 10.1016/j.plantsci.2015.04.017 26089147

[B13] Gellerman J. L. Anderson W. H. Schlenk H. (1976). Synthesis of anacardic acids in seeds of ginkgo biloba. Biochim. Biophys. Acta 431, 16–21. doi: 10.1016/0005-2760(76)90255-1 1268241

[B14] Gulec M. Iraz M. Yilmaz H. R. Ozyurt H. Temel I. (2006). The effects of ginkgo biloba extract on tissue adenosine deaminase, xanthine oxidase, myeloperoxidase, malondialdehyde, and nitric oxide in cisplatin-induced nephrotoxicity. Toxicol. Ind. Health 22, 125–130. doi: 10.1191/0748233705th255oa 16716042

[B15] Han X. He B. Xin Y. Xu M. Xu L. A. (2021). Full-length sequencing of ginkgo biloba l. reveals the synthesis of terpenoids during seed development. Ind. Crop Prod. 170, 113714. doi: 10.1016/j.indcrop.2021.113714

[B16] He B. Gu Y. C. Xu M. Wang J. W. Cao F. L. Xu L. A. (2015). Transcriptome analysis of ginkgo biloba kernels. Front. Plant Sci. 6, 113434. doi: 10.3389/fpls.2015.00819 PMC4593864 26500663

[B17] He B. Liu H. L. Han X. Cui P. Xu L. A. (2021). Multi-omics analysis of ginkgo biloba preliminarily reveals the co-regulatory mechanism between stilbenes and flavonoids. Ind. Crop Prod 167. doi: 10.1016/j.indcrop.2021.113434

[B18] Jiang L. Si Z. H. Li M. H. Zhao H. Fu Y. H. Xing Y. X.. (2017). H-1 NMR-based metabolomics study of liver damage induced by ginkgolic acid (15:1) in mice. J. Pharmaceut. BioMed. 136, 44–54. doi: 10.1016/j.jpba.2016.12.033 28063335

[B19] Kim Y. J. Lee O. R. Oh J. Y. Jang M. G. Yang D. C. (2014). Functional analysis of 3-hydroxy-3-methylglutaryl coenzyme a reductase encoding genes in triterpene saponin-producing ginseng. Plant Physiol. 165, 373–387. doi: 10.1104/pp.113.222596 24569845 PMC4012596

[B20] Kim D. Paggi J. M. Park C. Bennett C. Salzberg S. L. (2019). Graph-based genome alignment and genotyping with HISAT2 and HISAT-genotype. Nat. Biotechnol. 37, 907. doi: 10.1038/s41587-019-0201-4 31375807 PMC7605509

[B21] Knutzon D. S. Thompson G. A. Radke S. E. Johnson W. B. Knauf V. C. Kridl J. C. (1992). Modification of brassica seed oil by antisense expression of a stearoyl-acyl carrier protein desaturase gene. Proc. Natl. Acad. Sci. U.S.A. 89, 2624–2628. doi: 10.1073/pnas.89.7.2624 1557366 PMC48714

[B22] Kobayashi D. Yoshimura T. Johno A. Ishikawa M. Sasaki K. Wada K. (2015). Decrease in pyridoxal-5 '-phosphate concentration and increase in pyridoxal concentration in rat plasma by 4 '-o-methylpyridoxine administration. Nutr. Res. 35, 637–642. doi: 10.1016/j.nutres.2015.05.015 26092494

[B23] Lai C. P. Huang L. M. Chen L. O. Chan M. T. Shaw J. F. (2017). Genome-wide analysis of GDSL-type esterases/lipases in arabidopsis. Plant Mol. Biol. 95, 181–197. doi: 10.1007/s11103-017-0648-y 28840447

[B24] Langfelder P. Horvath S. (2008). WGCNA: an r package for weighted correlation network analysis. BMC Bioinf. 9, 559. doi: 10.1186/1471-2105-9-559 PMC2631488 19114008

[B25] Lee J. S. Cho Y. S. Park F. J. Kim J. Oh W. K. Lee H. S.. (1998). Phospholipase c gamma 1 inhibitory principles from the sarcotestas of ginkgo biloba. J. Nat. Prod 61, 867–871. doi: 10.1021/np970367q 9677265

[B26] Leistner E. Drewke C. (2010). Ginkgo biloba and ginkgotoxin. J. Nat. Prod 73, 86–92. doi: 10.1021/np9005019 20041670

[B27] Li B. Neumann E. K. Ge J. Y. Gao W. Yang H. Li P.. (2018). Interrogation of spatial metabolome of ginkgo biloba with high-resolution matrix-assisted laser desorption/ionization and laser desorption/ionization mass spectrometry imaging. Plant Cell Environ. 41, 2693–2703. doi: 10.1111/pce.13395 29966033

[B28] Liu H. L. Wang X. B. Wang G. B. Cui P. Wu S. G. Ai C.. (2021). The nearly complete genome of ginkgo biloba illuminates gymnosperm evolution. Nat. Plants 7, 748. doi: 10.1038/s41477-021-00933-x 34135482

[B29] Love M. I. Huber W. Anders S. (2014). Moderated estimation of fold change and dispersion for RNA-seq data with DESeq2. Genome Biol. 15, 550. doi: 10.1186/s13059-014-0550-8 25516281 PMC4302049

[B30] Mahadevan S. Park Y. (2008). Multifaceted therapeutic benefits of ginkgo biloba l.: chemistry, efficacy, safety, and uses. J. Food Sci. 73, R14–R19. doi: 10.1111/j.1750-3841.2007.00597.x 18211362

[B31] Mashek D. G. Bornfeldt K. E. Coleman R. A. Berger J. Bernlohr D. A. Black P.. (2004). Revised nomenclature for the mammalian long-chain acyl-CoA synthetase gene family. J. Lipid Res. 45, 1958–1961. doi: 10.1194/jlr.E400002-JLR200 15292367

[B32] Mei N. Guo X. Ren Z. Kobayashi D. Wada K. Guo L. (2017). Review of ginkgo biloba-induced toxicity, from experimental studies to human case reports. J. Environ. Sci. Health C Environ. Carcinog Ecotoxicol Rev. 35, 1–28. doi: 10.1080/10590501.2016.1278298 28055331 PMC6373469

[B33] Mindrebo J. T. Patel A. Kim W. E. Dayis T. D. Chen A. C. Bartholow T. G.. (2020). Gating mechanism of elongating beta-ketoacyl-ACP synthases. Nat. Commun. 11, 1727. doi: 10.1038/s41467-020-15455-x 32265440 PMC7138838

[B34] Muroi H. Kubo I. (1996). Antibacterial activity of anacardic acid and totarol, alone and in combination with methicillin, against methicillin-resistant staphylococcus aureus. J. Appl. Bacteriol 80, 387–394. doi: 10.1111/j.1365-2672.1996.tb03233.x 8849640

[B35] Nabavi S. M. Silva A. S. (2019). Nonvitamin and nonmineral nutritional supplements (San Diego, CA, United States: Academic Press, London, United Kingdom).

[B36] Nakano C. Ozawa H. Akanuma G. Funa N. Horinouchi S. (2009). Biosynthesis of aliphatic polyketides by type III polyketide synthase and methyltransferase in bacillus subtilis. J. Bacteriol 191, 4916–4923. doi: 10.1128/JB.00407-09 19465653 PMC2715739

[B37] Narnoliya L. K. Kaushal G. Singh S. P. Sangwan R. S. (2017). *De novo* transcriptome analysis of rose-scented geranium provides insights into the metabolic specificity of terpene and tartaric acid biosynthesis. BMC Genomics 18, 74. doi: 10.1186/s12864-016-3437-0 28086783 PMC5234130

[B38] Ou S. Su W. Liao Y. Chougule K. Agda J. R. A. Hellinga A. J.. (2019). Benchmarking transposable element annotation methods for creation of a streamlined, comprehensive pipeline. Genome Biol. 20, 275. doi: 10.1186/s13059-019-1905-y 31843001 PMC6913007

[B39] Pan W. Luo P. Fu R. Gao P. Long Z. Xu F.. (2006). Acaricidal activity against panonychus citri of a ginkgolic acid from the external seed coat of ginkgo biloba. Pest Manag Sci. 62, 283–287. doi: 10.1002/ps.1152 16475219

[B40] Penuelas J. Munne-Bosch S. (2005). Isoprenoids: an evolutionary pool for photoprotection. Trends Plant Sci. 10, 166–169. doi: 10.1016/j.tplants.2005.02.005 15817417

[B41] Pertea M. Pertea G. M. Antonescu C. M. Chang T. C. Mendell J. T. Salzberg S. L. (2015). StringTie enables improved reconstruction of a transcriptome from RNA-seq reads. Nat. Biotechnol. 33, 290. doi: 10.1038/nbt.3122 25690850 PMC4643835

[B42] Rodriguez M. Ringstad L. Schafer P. Just S. Hofer H. W. Malmsten M.. (2007). Reduction of atherosclerotic nanoplaque formation and size by ginkgo biloba (EGb 761) in cardiovascular high-risk patients. Atherosclerosis 192, 438–444. doi: 10.1016/j.atherosclerosis.2007.02.021 17397850

[B43] Sagar M. Pandey N. Qamar N. Singh B. Shukla A. (2015). Domain analysis of 3 keto acyl-CoA synthase for structural variations in vitis vinifera and oryza brachyantha using comparative modelling. Interdiscip Sci. 7, 7–20. doi: 10.1007/s12539-013-0017-8 25239516

[B44] Sasaki Y. Noguchi T. Yamamoto E. Giddings J. C. Ikeda K. Yamori Y.. (2002). Effects of ginkgo biloba extract (EGb 761) on cerebral thrombosis and blood pressure in stroke-prone spontaneously hypertensive rats. Clin. Exp. Pharmacol. 29, 963–967. doi: 10.1046/j.1440-1681.2002.03761.x 12366386

[B45] Schmidt A. Gershenzon J. (2007). Cloning and characterization of isoprenyl diphosphate synthases with farnesyl diphosphate and geranylgeranyl diphosphate synthase activity from Norway spruce (Picea abies) and their relation to induced oleoresin formation. Phytochemistry 68, 2649–2659. doi: 10.1016/j.phytochem.2007.05.037 17624381

[B46] Schroder J. Schroder G. (1990). Stilbene and chalcone synthases: related enzymes with key functions in plant-specific pathways. Z Naturforsch. C J. Biosci. 45, 1–8. doi: 10.1515/znc-1990-1-202 2184816

[B47] Shepard S. McCreary M. Fedorov A. (2009). The peculiarities of large intron splicing in animals. PloS One 4, e7853. doi: 10.1371/journal.pone.0007853 19924226 PMC2773006

[B48] Thevenot E. A. Roux A. Xu Y. Ezan E. Junot C. (2015). Analysis of the human adult urinary metabolome variations with age, body mass index, and gender by implementing a comprehensive workflow for univariate and OPLS statistical analyses. J. Proteome Res. 14, 3322–3335. doi: 10.1021/acs.jproteome.5b00354 26088811

[B49] Thissen D. Steinberg L. Kuang D. (2002). Quick and easy implementation of the benjamini-hochberg procedure for controlling the false positive rate in multiple comparisons. J. Educ. Behav. Stat. 27, 77–83. doi: 10.3102/10769986027001077

[B50] van Beek T. A. (2002). Chemical analysis of ginkgo biloba leaves and extracts. J. Chromatogr A 967, 21–55. doi: 10.1016/S0021-9673(02)00172-3 12219929

[B51] van Beek T. A. (2005). Ginkgolides and bilobalide: their physical, chromatographic and spectroscopic properties. Bioorgan Med. Chem. 13, 5001–5012. doi: 10.1016/j.bmc.2005.05.056 15993092

[B52] Wang D. Zhang Y. Zhang Z. Zhu J. Yu J. (2010). KaKs_Calculator 2.0: a toolkit incorporating gamma-series methods and sliding window strategies. Genomics Proteomics Bioinf. 8, 77–80. doi: 10.1016/S1672-0229(10)60008-3 PMC5054116 20451164

[B53] Yang Y. F. Li Y. Wang J. H. Sun K. Tao W. Y. Wang Z. Z.. (2017). Systematic investigation of ginkgo biloba leaves for treating cardio-cerebrovascular diseases in an animal model. ACS Chem. Biol. 12, 1363–1372. doi: 10.1021/acschembio.6b00762 28333443

[B54] Yang X. M. Wang Y. F. Li Y. Y. Ma H. L. (2014). Thermal stability of ginkgolic acids from ginkgo biloba and the effects of ginkgol C17:1 on the apoptosis and migration of SMMC7721 cells. Fitoterapia 98, 66–76. doi: 10.1016/j.fitote.2014.07.003 25016955

[B55] Ye J. B. Mao D. Cheng S. Y. Zhang X. Tan J. P. Zheng J. R.. (2020). Comparative transcriptome analysis reveals the potential stimulatory mechanism of terpene trilactone biosynthesis by exogenous salicylic acid in ginkgo biloba. Ind. Crop Prod 145, 112104. doi: 10.1016/j.indcrop.2020.112104

[B56] Yu D. Y. Xu F. C. Zeng J. Zhan J. X. (2012). Type III polyketide synthases in natural product biosynthesis. IUBMB Life 64, 285–295. doi: 10.1002/iub.1005 22362498

[B57] Zheng X. Li P. Lu X. (2019). Research advances in cytochrome P450-catalysed pharmaceutical terpenoid biosynthesis in plants. J. Exp. Bot. 70, 4619–4630. doi: 10.1093/jxb/erz203 31037306

[B58] Zhou J. M. Ibrahim R. K. (2010). Tricin-a potential multifunctional nutraceutical. Phytochem. Rev. 9, 413–424. doi: 10.1007/s11101-009-9161-5

[B59] Zhou Z. Zheng S. (2003). The missing link in ginkgo evolution. Nature 423, 821–822. doi: 10.1038/423821a 12815417

